# Multifaceted mitochondrial as a novel therapeutic target in dry eye: insights and interventions

**DOI:** 10.1038/s41420-024-02159-0

**Published:** 2024-09-06

**Authors:** Weijie Ouyang, Dan Yan, Jiaoyue Hu, Zuguo Liu

**Affiliations:** 1 Xiamen University affiliated Xiamen Eye Center, Fujian Provincial Key Laboratory of Ophthalmology and Visual Science, Fujian Engineering and Research Center of Eye Regenerative Medicine, Eye Institute of Xiamen University, School of Medicine of Xiamen University, Xiamen, Fujian, China; Department of Ophthalmology, the Affiliated Hospital of Guizhou Medical University, Guiyang, Guizhou China; 2 Xiamen University affiliated Xiamen Eye Center, Fujian Provincial Key Laboratory of Ophthalmology and Visual Science, Fujian Engineering and Research Center of Eye Regenerative Medicine, Eye Institute of Xiamen University, School of Medicine of Xiamen University, Xiamen, Fujian China; 3 Xiamen University affiliated Xiamen Eye Center, Fujian Provincial Key Laboratory of Ophthalmology and Visual Science, Fujian Engineering and Research Center of Eye Regenerative Medicine, Eye Institute of Xiamen University, School of Medicine of Xiamen University, Department of Ophthalmology of Xiang’an Hospital of Xiamen University, Xiamen, Fujian China; 4 Xiamen University affiliated Xiamen Eye Center, Fujian Provincial Key Laboratory of Ophthalmology and Visual Science, Fujian Engineering and Research Center of Eye Regenerative Medicine, Eye Institute of Xiamen University, School of Medicine of Xiamen University, Department of Ophthalmology of Xiang’an Hospital of Xiamen University, Xiamen, Fujian, China; Department of Ophthalmology, the First Affiliated Hospital of University of South China, University of South China, Hengyang, Hunan China

**Keywords:** Eye diseases, Biomarkers

## Abstract

Dry eye, recognized as the most prevalent ocular surface disorder, has risen to prominence as a significant public health issue, adversely impacting the quality of life for individuals across the globe. Despite decades of extensive research into the chronic inflammation that characterizes dry eye, the intricate mechanisms fueling this persistent inflammatory state remain incompletely understood. Among the various cellular components under investigation, mitochondria—essential for cellular energy production and homeostasis—have attracted increasing attention for their role in dry eye pathogenesis. This involvement points to mechanisms such as oxidative stress, apoptosis, and sustained inflammation, which are central to the progression of the disease. This review aims to provide a thorough exploration of mitochondrial dysfunction in dry eye, shedding light on the critical roles played by mitochondrial oxidative stress, apoptosis, and mitochondrial DNA damage. It delves into the mechanisms through which diverse pathogenic factors may trigger mitochondrial dysfunction, thereby contributing to the onset and exacerbation of dry eye. Furthermore, it lays the groundwork for an overview of current therapeutic strategies that specifically target mitochondrial dysfunction, underscoring their potential in managing this complex condition. By spotlighting this burgeoning area of research, our review seeks to catalyze the development of innovative drug discovery and therapeutic approaches. The ultimate goal is to unlock promising avenues for the future management of dry eye, potentially revolutionizing treatment paradigms and improving patient outcomes. Through this comprehensive examination, we endeavor to enrich the scientific community’s understanding of dry eye and inspire novel interventions that address the underlying mitochondrial dysfunctions contributing to this widespread disorder.

## Facts

- Dry eye is the most common ocular surface disorder, characterized by chronic inflammation.
- Mitochondrial dysfunction, including oxidative stress, apoptosis, and sustained inflammation, play a critical role in the progression of dry eye.
- Therapeutic strategies targeting mitochondrial dysfunction have become a burgeoning area of research in the treatment of dry eye.

## Open questions

- What are the exact mechanisms driving the persistent chronic inflammation in dry eye?
- How do various pathogenic factors trigger mitochondrial dysfunction, and what is the relationship between these factors and the onset and severity of dry eye?
- What role does mitochondria dysfunction play in dry eye?
- How can we effectively target mitochondria dysfunction target mitochondrial dysfunction for therapeutic purposes?

## Introduction

Dry eye (DE), identified as one of the most prevalent ocular surface diseases, manifests through tear film instability, hyperosmolarity, chronic inflammation, and a spectrum of ocular discomfort symptoms [1, 2]. The global prevalence of DE is estimated to range from 5% to 50%, positioning it as a significant public health issue worldwide [3]. Chronic inflammation plays a crucial role in the pathology of DE, where prolonged tear film instability and tear hyperosmolarity lead to epithelial cell damage [4]. Such damage triggers the release of pro-inflammatory mediators, subsequently activating antigen-presenting cells (APCs) and fostering the maturation of CD4^+^ T cells. These CD4^+^ T cells evolve into Th1 and Th17 subtypes, secreting IFN-γ and IL-17, respectively, further exacerbating epithelial cell damage and perpetuating the harmful “damage-inflammation” cycle in DE [5]. However, the initial factors triggering this chronic inflammatory cascade remain unclear, suggesting that the pathogenesis of DE involves more than just inflammatory disruptions [6].

In this context, mitochondria, serving as the cellular energy metabolism center, play vital roles in essential cellular functions such as lipid metabolism, ion balance, calcium signaling, and apoptosis [7]. Maintaining mitochondrial homeostasis is critical for preserving corneal epithelial integrity, defending against oxidative damage and inflammation, and promoting corneal epithelial migration and repair [8]. Notably, various etiological factors, including environmental stress, blue light exposure, autoimmune diseases (e.g., Sjögren’s syndrome), metabolic disorders (e.g., diabetes), and the natural aging process [3], have been linked to mitochondrial dysfunction in the development of DE. Increasing empirical evidence supports the significant role of mitochondrial dysfunction in DE [9–13], including mitochondrial oxidative stress [14], mitochondrial apoptosis [15], mitochondrial DNA (mtDNA) damage [16] and so on. Interestingly, the antioxidant agents, targeting mitochondrial dysfunction, has shown promising therapeutic effects in patients with DE [17], highlighting the potential of mitochondrial modulation as a treatment approach.

Therefore, this review meticulously examines the contribution of mitochondrial dysfunction to DE, delves into the mechanisms by which various etiological factors induce mitochondrial dysfunction leading to DE, and synthesizes current insights into the treatment of mitochondrial dysfunction in DE. We conducted a comprehensive literature search using PubMed, Web of Science, and Scopus. The keywords included “dry eye,” “mitochondrial dysfunction,” “reactive oxygen species (ROS),” “mitochondrial apoptosis,” “mitochondrial DNA (mtDNA),” and “therapy.” We included articles published in peer-reviewed journals that focused on mitochondrial dysfunction in DE, discussed therapeutic strategies targeting this dysfunction, and involved human subjects, animal models, or in vitro studies relevant to the pathophysiology and treatment of DE. We excluded articles not available in English, studies not directly related to mitochondrial dysfunction and DE, and reviews or meta-analyses lacking new empirical data. The initial search yielded numerous articles, which were then screened by titles and abstracts to identify those meeting the inclusion criteria. Full texts of potentially relevant articles were retrieved and assessed for eligibility, and reference lists of selected articles were reviewed for additional studies. Data from the included studies were extracted independently by two authors, with discrepancies resolved through discussion. The extracted data were qualitatively synthesized to provide a comprehensive overview of the current understanding of mitochondrial dysfunction in DE disease and potential therapeutic approaches. The aim is to elucidate novel therapeutic strategies and enhance understanding of the complex etiology of DE. By integrating the latest literature, this review hopes to advance the discussion on mitochondria-centered interventions, positioning mitochondrial homeostasis as a novel and critical target in the comprehensive management of DE.

## Mitochondria

Mitochondria, universally acknowledged as the cellular powerhouses, harbor a distinctive dual membrane structure, complete with cristae and a unique circular genome. These organelles are not merely confined to energy production; their functions extend to calcium regulation, amino acid metabolism, and the orchestration of antiviral, apoptotic, and DNA signaling pathways [18]. At the heart of these diverse roles lies their critical function as the metabolic hub for cellular energy generation. The electron transport chain (ETC), located within the inner mitochondrial membrane (IMM), plays a pivotal role in ATP synthesis through oxidative phosphorylation (OXPHOS), supplying up to 90% of a cell’s energy needs [19, 20].

The vitality of mitochondria extends across various physiological systems, emphasizing the necessity of an intact and fully functional mitochondrial architecture for efficient energy production [21]. However, the complex structure of mitochondria makes them particularly vulnerable to external stressors [22, 23]. Such environmental challenges can compromise mitochondrial integrity, leading to redox imbalances and disruptions in their functional capabilities [24]. In the context of DE, our recent research has shed light on the profound impact of desiccation stress on mitochondrial health [25]. It was observed exacerbated mitochondrial membrane permeability and mtDNA release in corneal epithelial cells from DE mouse models, underlining the direct consequences of such stress on both mitochondrial and broader cellular molecular frameworks.

### Mitochondrial microstructure and fundamental functions

Mitochondria are essential organelles responsible for various crucial functions within cells, including energy production, metabolism, and signaling [26]. A closer examination of the microstructure and fundamental functions of mitochondria highlights the intricate organization and roles of different components within these organelles. The outer membrane of mitochondria contains porins that play a vital role in facilitating ATP synthesis, protein transport, and energy transfer. Among these porins, Voltage-Dependent Anion Channels (VDAC) and the Translocase of the outer membrane (Tom) serve as critical gateways for molecules entering and exiting the mitochondria, thereby regulating essential processes [27, 28]. Within the inner membrane of mitochondria, structures known as cristae are enriched with enzymes that are crucial for ATP synthesis. These enzymes include ATP synthase, which is responsible for generating ATP, and components of the ETC, which play a key role in the process of OXPHOS to produce ATP [18, 29]. The mitochondrial matrix, a soluble component enclosed by the inner membrane, acts as a biochemical dynamo within the mitochondria. It houses essential components such as mtDNA, RNA, and various enzymes that are vital for lipid metabolism and the tricarboxylic acid (TCA) cycle, also known as the citric acid cycle or Krebs cycle [30, 31]. These components work together to carry out metabolic processes that result in the production of energy in the form of ATP. Overall, the microstructure and functions of mitochondria highlight the coordinated interplay of different components and processes that are essential for cellular energy production, metabolism, and overall cellular function.

### Mitochondrial genetics and dynamics

The semi-autonomous nature of mitochondria, characterized by their own genetic material and dynamic behavior, plays a crucial role in cellular health and disease [32, 33]. mtDNA is a unique, histone-free, double-stranded circular molecule that is particularly vulnerable to mutations and damage induced by ROS [34, 35]. This susceptibility positions mitochondria as primary victims of oxidative stress, emphasizing the importance of mitochondrial integrity in cellular function. Mitochondrial dynamics, which include the processes of mitochondrial fission and fusion, are critical for maintaining cellular metabolism, supporting embryonic development, and regulating apoptosis [36, 37]. These dynamics are orchestrated by specific proteins: Mnf1, Mnf2, and OPA1 facilitate fusion, while Drp1, Fis1, Mff, and MiD49/51 are responsible for fission. The delicate balance between these two processes ensures efficient energy production and contributes to the overall health of the cell. Dysregulation of mitochondrial dynamics can significantly impair energy production efficiency, leading to detrimental effects on cellular aging [38]. This is largely due to the intimate connection between ROS production and mitochondrial integrity. As mitochondria are the main site of ROS generation during energy production, any disruption in their function or structure can exacerbate ROS levels, further damaging mtDNA and impairing mitochondrial function [39, 40]. This vicious cycle highlights the profound influence of ROS on mitochondrial energy production, consumption, and lifecycle, thereby closely linking mitochondrial dynamics to cellular bioenergetics and aging.

In essence, the intricate relationship between mitochondrial structure, function, and dynamics underscores the vital role of mitochondria in determining cellular health and susceptibility to disease. Understanding this relationship is key to unraveling the complex mechanisms underlying various pathologies and developing targeted interventions to preserve or restore mitochondrial function, potentially mitigating the effects of aging and disease.

**Fig. 1:**
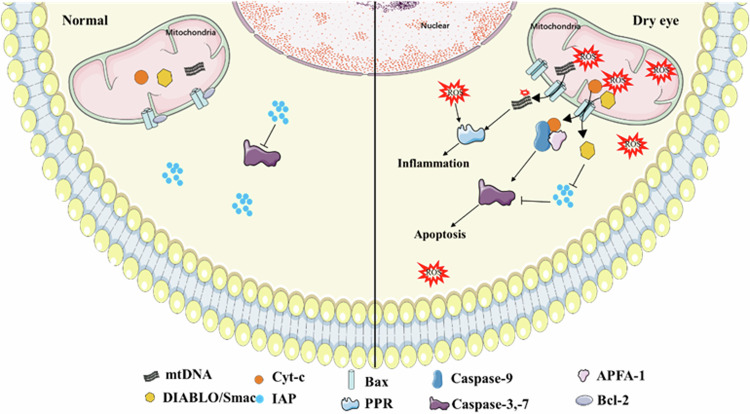
Role of mitochondrial dysfunction in DE. The risk factors for DE-induced mitochondrial dysfunction. Mitochondrial dysfunction increases intracellular ROS accumulation, activating the NLRP3 and NF-kB signaling pathways and the release of downstream inflammatory cytokines. Mitochondrial dysfunction increases mitochondrial permeability by inducing the expression of Bax. Cyt-c and DIABLO/Smac are released from mitochondria to the cytoplasm. Cyt-c binds and activates APAF-1 to form apoptotic bodies and activates Caspase-9- and Caspase-3-7-dependent apoptosis. DIABLO/Smac activates apoptosis by blocking IAP. Additionally, mtDNA can be released into the cytoplasm and recognized by pattern recognition receptors to initiate the inflammatory response.

## Known Roles of mitochondrial dysfunction in DE (Fig. 1)

### Mitochondrial oxidative stress in DE

Mitochondria stand as the principal metabolic centers for cellular energy, generating ATP through OXPHOS to meet the cell’s energy needs [41]. A notable byproduct of this process is the generation of ROS, primarily produced by mitochondria during normal aerobic respiration [42, 43]. Approximately 2% of electrons escape from the ETC, leading to ROS formation within the mitochondria [19]. Accumulation of intracellular ROS, stemming from mitochondrial dysfunction, can provoke cellular oxidative damage [44].

DE, a multifactorial disease, is characterized by oxidative stress within the cornea, frequently accompanied by ocular surface inflammation [45]. Both in vivo and in vitro models of DE have demonstrated ROS accumulation in corneal epithelial cells [14]. This overproduction of ROS leads to lipid peroxidation, evidenced by increased levels of toxic byproducts such as 4-hydroxy-2-nonenal (4-HNE) and malondialdehyde. The resultant imbalance between oxidase and antioxidant enzymes is marked by heightened oxidant activity of heme oxygenase 1 (HO-1) and cyclooxygenase 2 (COX2), alongside diminished antioxidant activity of superoxide dismutase 1 (SOD1) and glutathione peroxidase 1 (GPX1) [46, 47]. The mev-1 gene, encoding Cyt-1—a key component of complex II in the mitochondrial ETC—has been implicated in excessive oxidative stress associated with ocular surface epithelial damage and reduced tear production in a Tet-mev-1 conditional transgenic mouse model. Histopathological analysis revealed significant monocyte infiltration and fibrosis in the lacrimal glands of these mice, illustrating the link between mitochondrial oxidative damage and lacrimal gland dysfunction in DE [48, 49]. In addition to the primary risk factors discussed, other lifestyle-related factors such as sleep deprivation [50], mental disorders [51, 52], and unhealthy lifestyles [53] also play significant roles in the pathogenesis of dry eye disease. Sleep deprivation has been shown to increase oxidative stress and inflammatory markers, thereby worsening dry eye symptoms. Mental health conditions like depression and anxiety can alter tear production through autonomic dysregulation and medication side effects. Unhealthy lifestyle choices, including poor diet, excessive alcohol consumption, and smoking, contribute to oxidative stress and inflammation, further impacting ocular surface health. Recognizing and addressing these additional risk factors is crucial for comprehensive management of dry eye disease.

In patients with DE (both Sjögren and non-Sjögren related), elevated concentrations of lipid peroxides (LPO), myeloperoxidase (MPO), 4-HNE, MDA, and hexanoyl-lysine (HEL) have been observed in tears [54–56], with increased levels of LPO, xanthine oxidoreductase/xanthine oxidase, nitric oxide synthase (NOS2, NOS3), 4-HNE, MDA, and ROS in the conjunctival epithelium [55–60]. These oxidative markers correlate with DE severity, with variations in antioxidant enzyme expression between Sjögren and non-Sjögren related DE [61, 62].

Oxidative stress-induced ROS can activate inflammatory pathways, including NLRP3, ASC, and caspase-1, leading to increased IL-1β expression and inflammation. N-acetyl-1-cysteine (NAC) has been shown to inhibit IL-1β accumulation and expression [59, 63]. Furthermore, ROS directly triggers the NF-κB pathway [64], promoting the release of pro-inflammatory cytokines such as TNF-α, IL-6, and IL-1β [65]. Reducing ROS accumulation has been demonstrated to inhibit NFκB translocation and decrease the expression of these cytokines [66], underscoring the pivotal role of oxidative stress in the pathogenesis of DE.

### Mitochondrial apoptosis in DE

Mitochondrial apoptosis represents a crucial intrinsic pathway of cell death, predominantly regulated by the B-cell lymphoma 2 (Bcl-2) family proteins [67]. This family bifurcates into anti-apoptotic and pro-apoptotic groups, exemplified by Bcl-2 and Bax, respectively [68]. Located on the outer mitochondrial membrane (OMM), Bcl-2 maintains the permeability of the OMM. Upon activation of the intrinsic apoptotic pathway, expression of Bcl-2 is suppressed while that of Bax increases, leading to the opening of the permeability transition pores (PTP), enhanced mitochondrial membrane permeability, loss of mitochondrial membrane potential, and the release of apoptotic proteins such as cytochrome C (Cyt-c) and DIABLO/Smac into the cytosol. Cyt-c binds and activates APAF-1, forming the apoptosome, which in turn activates Caspase-9. Caspase-9 triggers a caspase cascade, further activating Caspase-3 and Caspase-7 to induce apoptosis. DIABLO/Smac enhances the apoptotic signal by blocking the inhibitors of apoptosis proteins (IAPs), thus activating Caspase-3, Caspase-7, and Caspase-9 [69, 70].

Extensive research has demonstrated an increase in TUNEL-positive corneal and conjunctival epithelial cells both in vivo and in vitro in DE conditions, indicating an upsurge in apoptotic cell death [71, 72]. Models of DE have shown decreased expression of Bcl-2 and increased levels of Bax, Cyt-c, and Caspase-3 [15, 73]. IFN-γ appears to play a pivotal role in mediating apoptosis within the DE context. Increased apoptotic cells, including Caspase-8 and TUNEL-positive cells, were observed in the conjunctiva of DE mice [74]. However, IFN-γ knockout mice exhibited resistance to apoptosis induced by DE. The induction of apoptosis and the production and activity of Caspase-9 in IFN-γ knockout mice subjected to DE conditions through subconjunctival injection of IFN-γ underscore the critical role of IFN-γ in regulating conjunctival cell apoptosis via dual apoptotic pathways in DE models [74]. Interestingly, IFN-γ was found to promote only the extrinsic apoptotic pathway in the cornea within the dry eye model [75].

The therapeutic significance of inhibiting the mitochondrial apoptotic pathway in dry eye treatment is underscored by studies on Cyclosporin A (CsA) [76, 77]. An increase in Caspase-3 and BAX, along with a decrease in Bcl-2, was observed in dry eye conditions. However, CsA treatment reversed these changes, highlighting the potential of targeting the mitochondrial apoptotic pathway as a therapeutic strategy in dry eye management.

### Mitochondria as a platform for innate immune signaling

The human mitochondrial genome was first sequenced in 1981, containing 16,569 base pairs [78]. These genes encode various ribosomal RNAs and proteins related to the mitochondrial respiratory chain, such as Cyt-c oxidase subunits I, II, and III, as well as ATPase subunit 6. Mitochondria contain multiple copies of mtDNA, ranging from 1 to 10 copies [79]. The maintenance of mtDNA copy number facilitates mitochondrial division, allowing damaged materials to segregate into smaller mitochondria, termed as “ρ0” cells, with reduced membrane potential and increased ROS levels [80].

Due to deficiencies in mtDNA repair mechanisms, lack of histone protection, and the proximity of mtDNA to the IMM where ROS are generated, mtDNA is highly susceptible to oxidative damage [81]. ROS originating from mitochondria are considered a major cause of mtDNA damage. Additionally, factors such as UV radiation and aging exacerbate mtDNA damage, leading to diseases, particularly ocular conditions like dry eye and age-related macular disease (AMD) [82–84].

Several studies have reported an increase in 8-hydroxy-2′-deoxyguanosine (8-OHdG), a biomarker of oxidative DNA damage, in mtDNA in both in vivo [85, 86] and in vitro [46, 47] dry eye models. Oxidative mtDNA damage elevates 8-OHdG expression, potentially leading to NLRP3/NLRP6 imbalance, Caspase-1 activation, and release of IL-1 and IL-18 [16]. These findings suggest that oxidative mtDNA may serve as a damage-associated molecular pattern (DAMP) triggering inflammatory responses in dry eye. Elevated oxidative stress levels in mitochondria and the release of mtDNA have been observed in patients with Sjögren’s syndrome [11]. Autoimmune diseases like Sjögren’s syndrome, systemic lupus erythematosus, and rheumatoid arthritis are often associated with increased levels of circulating DNA, with the disease index of Sjögren’s syndrome correlating with circulating DNA levels [87]. Recent research has highlighted the accumulation of free DNA in the circulation of Sjögren’s syndrome patients, along with significant accumulation of mtDNA in exocrine glands, associated with NLRP3 inflammasomes and IL-18 [88]. This provides new biomarkers and therapeutic targets for Sjögren’s syndrome. Released mtDNA can be recognized by pattern recognition receptors (PRRs) such as NLRP3, TLR9, cGAS/STING, and ZBP1 to initiate inflammatory responses [11]. Given the invasive nature of Sjögren’s syndrome involving exocrine glands and the significant accumulation of mtDNA in salivary glands, it is likely that mtDNA accumulation also occurs in lacrimal glands of Sjögren’s syndrome patients. Further research is needed to confirm the role of mtDNA in lacrimal gland inflammation and dysfunction. Our latest research has revealed that in dry eye-induced ocular surface damage, the release of mtDNA from corneal epithelial cells activates the cGAS-STING signaling pathway [25]. This discovery sheds light on the connection between mitochondrial dysfunction and the development of ocular inflammation, offering a new perspective for studying the pathophysiological processes of ocular inflammatory responses.

**Fig. 2:**
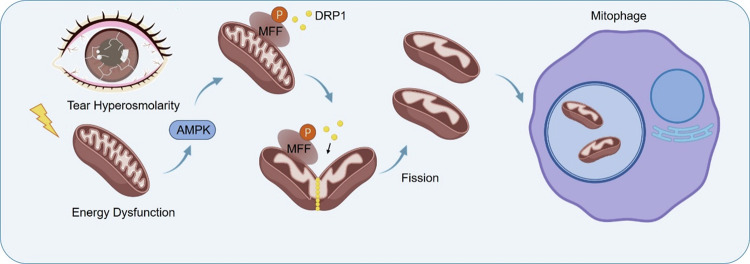
Mitophagy. In cases of DE syndrome, the condition of tear hyperosmolarity acts as a critical trigger for mitochondrial reactive oxygen species (mtROS) generation and subsequent cellular energy dysfunction. This process potentially leads to the activation of the AMP-activated protein kinase/Mitochondrial Fission Factor (AMPK/MFF) pathway in Human Corneal Epithelial Cells (HCECs). Once activated, MFF plays a pivotal role in recruiting DRP1—a cytoplasmic protein—to the OMM. This recruitment is a key step in mediating both mitochondrial fission and mitophagy, processes essential for maintaining cellular health and function under stress conditions.

### Mitophagy (Fig. 2)

The balance of mitochondrial morphology and dynamics is crucial for maintaining mitochondrial homeostasis [89]. Mitochondrial dynamics involve the control of mitochondrial morphology through fission and fusion processes. Mitochondrial fragmentation or fission is a common response to stress, affecting the energy status of mitochondria and consequently influencing organelle fate [78]. Excessive fission can lead to mitochondrial damage, potentially harming cardiac, pancreatic, and neuronal cells [90]. Damaged mitochondria need to be repaired or removed to preserve cellular homeostasis. Mitophagy, a highly regulated multistep process, selectively degrades damaged or dysfunctional mitochondria. Mitochondrial fission is a prerequisite for mitophagy, as fission can promote mitophagy. Moderate autophagy helps remove damaged organelles from cells and maintain cellular homeostasis, while excessive autophagy can lead to autophagic cell death. Studies [14] using a cellular model of hyperosmotic stress have shown widespread mitochondrial fission and mitophagy in corneal epithelial cells under such conditions. Metabolic disturbances occur under hyperosmotic stress, leading to the activation of AMPK. Furthermore, AMPK activation phosphorylates MFF, which recruits DRP1 to the OMM, mediating mitochondrial fission and mitophagy. Inhibiting MFF to suppress mitochondrial fission can reverse the recurrence of dry eye syndrome.

**Fig. 3:**
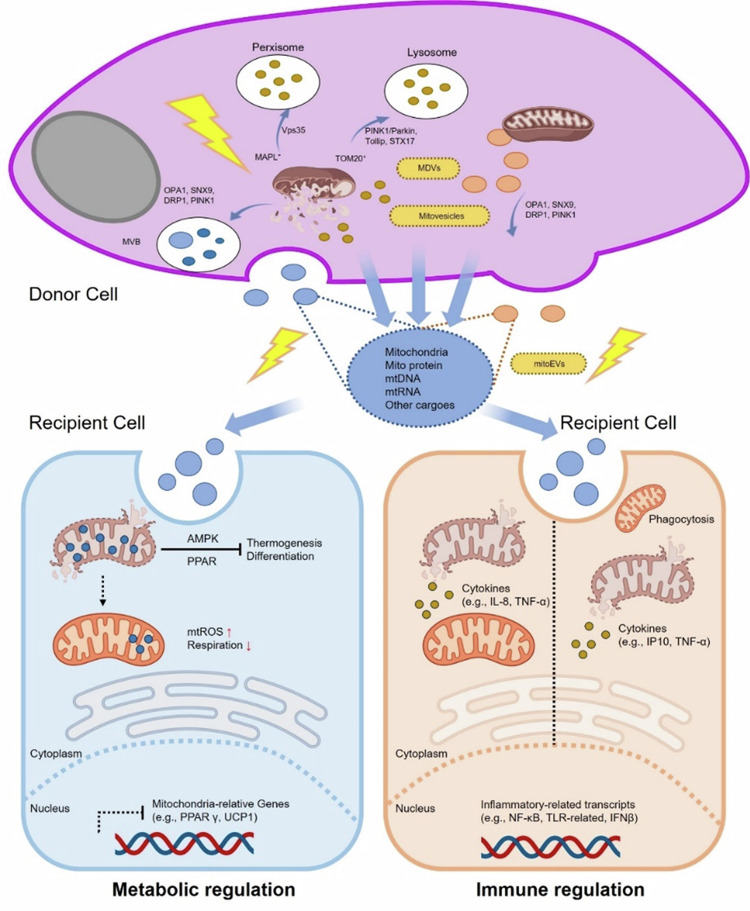
Sorting mechanisms and biological effects of mitoEVs. Potential Sorting Mechanisms in Donor Cells. The formed MDVs can be sorted into lysosomes via PINK1/Parkin, Tollip, or STX17 pathways; into peroxisomes through Vps35 and MAPL; and into the extracellular space via OPA1, SNX9, DRP1, or PINK1. In certain cases, MDVs budding from mitochondria may merge into multivesicular bodies (MVBs), subsequently being released into the extracellular space as mitochondrial EVs. Moreover, mitoEVs encompass novel EV subtypes, mitochondrial vesicles, and other potential pathways involved in mitoEV biogenesis that require further categorization. Diverse effects of these EVs in target cells. These EVs may exhibit metabolic regulatory functions, such as disruptions in mitochondrial biogenesis (e.g., AMPK, PGC1), mitochondrial respiration, and mtROS production, thereby mediating the receptor cell’s phenotypic changes (e.g., differentiation, apoptosis). MitoEVs also possess immunomodulatory functions, including the induction of pro-inflammatory signals (e.g., TLR and STING), cytokine release, IFN responses, and phagocytic activities in immune cells.

### Mitochondrial transfer (Fig. 3)

In recent years, scholars have discovered that cells can form tunneling nanotubes (TNTs) to facilitate intercellular communication, enabling mitochondrial transfer between different cells to maintain metabolic balance [91]. This transfer phenomenon has been reported in many systems, including the nervous and respiratory systems. Astrocytes can transfer mitochondria to neurons, potentially providing neuroprotection and promoting neural recovery after stroke [92]. In an ischemic stroke rat model, pretreatment with melatonin for mitochondrial transfer has been found to reduce the area of brain infarction [93]. These findings suggest that intercellular mitochondrial transfer can occur under pathological conditions, where damaged mitochondria can partially restore the function of recipient cells. Previous studies have indicated that mitochondrial transport is a common intercellular mechanism that helps repair mitochondrial damage within cells [94]. The transfer of mitochondria between healthy corneal epithelial cells is typically inefficient. Hypertonic therapy can enhance the formation of TNTs and promote human mitochondrial transfer. This aligns with previous work by many researchers, indicating that stress can facilitate intercellular mitochondrial transfer, possibly related to the ability of damaged cells to emit “SOS” signals to promote such transfer. In general, mitochondrial damage serves as the primary trigger for TNT-based mitochondrial transfer [95]. However, whether this transfer contributes to the worsening damage and vicious cycle in dry eye syndrome remains unexplored. A study exploring the therapeutic effects of mesenchymal stem cells on dry eye syndrome revealed that these cells primarily improve corneal epithelial cells through mitochondrial transfer [96]. This indirectly demonstrates that mitochondrial transfer can occur in dry eyes. Live-cell imaging showing vesicles and mitochondria labeled with DiO transferring from one TM cell to another was firstly described in 2017, proving that normal and glaucomatous TM cells communicate with each other through TNTs. Literature reports suggest that vascular endothelial growth factor and hypoxia-inducible factor-1α signals can be transmitted through TNTs between malignant ovarian cancer cells and human vascular endothelial cells to write vascular endothelial growth factors [95].

Therefore, it remains to be seen whether the transfer of damaged corneal epithelial cell mitochondria leads to further damage in relatively normal cells through inflammatory promotion, oxidative stress, and related genes. Alternatively, could the transfer of mitochondria from damaged corneal epithelial cells to immune cells trigger an inflammatory response, exacerbating dry eye symptoms? These questions point towards potential future research directions.

### Mitochondria OXPHOS

Mitochondrial metabolic events have been demonstrated to exert profound influences on immunological competence [97]. The primary role of mitochondria is the generation of ATP through the OXPHOS process. This metabolic activity within mitochondria significantly impacts immune responses [98, 99]. In the context of dry eye disease, innate immune responses prominently feature macrophages and dendritic cells (DCs) as key inflammatory immune cells [100, 101]. Macrophages are categorized into M1 and M2 cells, with the former associated with pro-inflammatory responses and the latter exerting regulatory functions [102]. DCs are differentiated into myeloid cells (DC1) and lymphoid-like cells (DC2). In dry eye conditions, DC2 cells primarily participate in immune regulation. The interplay between these cells and the expression of various cytokines, including interleukins and tumor necrosis factors, leads to sustained elevation of inflammatory receptor levels, culminating in dry eye syndrome [103]. Metabolic reprogramming is pivotal for dendritic cell activation and maturation. Literature indicates that active oxidative phosphorylation in mitochondria is associated with immature or tolerant DCs across multiple systemic contexts [104]. However, studies exploring the metabolic states of DCs in dry eye disease are lacking. This gap presents a novel direction for research into the pathogenic mechanisms underlying dry eye disease.

Furthermore, adaptive immunity plays a crucial role in this condition [105]. During the adaptive immune phase, the production of antigen-specific T cells in regional lymph nodes, followed by their migration to the ocular surface under stress, initiates a pathological cycle of immune responses. This includes the proliferation and expansion of T cells at the ocular surface, causing damage, reactivation of acute pro-inflammatory innate responses, and, as immune regulation is lost, a vicious cycle of pathological immune reactions [106, 107]. The quality, potential activity, and alterations in mitochondrial activity are crucial for regulating the development, fate, and function of T cells [108, 109]. Multiple studies have indicated that immunological signaling and metabolic stress directly contribute to the regulation of T cell fate and function within the tumor microenvironment, with mitochondria acting as the orchestrative hub for these molecular processes [110, 111]. Nevertheless, despite the acknowledged pathogenic role of lymphocytes in dry eye disease, the impact of mitochondrial function within these lymphocytes remains unexplored.

## Risk factors for dry eye are related to mitochondrial dysfunction

### Environment-related risk factors

#### Desiccation stress

Desiccation stress plays a crucial role in the development of dry eye. Conditions such as low humidity, high wind speed, or high temperature can easily cause excessive evaporation of tears, leading to tear hyperosmolarity. According to the findings presented in Table 1, hyperosmotic stress alters the levels of mitochondrial calcium in corneal epithelial cells, causes depolarization of the mitochondrial membrane potential, and reduces ATP synthesis, resulting in mitochondrial dysfunction [9]. This dysfunction, in turn, leads to the accumulation of ROS in corneal epithelial cells and an increase in the levels of 4-HNE and 8-OHDG [46]. On one hand, the upregulation of Bax expression results in an increase in mitochondrial membrane permeability [15]. On the other hand, oxidative mtDNA is released into the cytoplasm due to the elevated MMP, triggering innate immune responses, such as NLRP3 activation, and subsequently leading to the upregulation of inflammatory factors like IL-1 [16]. Additionally, the accumulated ROS can facilitate the translocation of NFκB, which induces the expression of pro-inflammatory cytokines such as TNF-α, IL-6, and IL-1β [66].

**Table 1:** Risk factors of DE are related to mitochondrial dysfunction.

Risk factors	Oxidative stress	Mitochondrial apoptosis	Mitochondrial DNA	Others	Ref.
Environment-related risk factors					
Desiccation stress	ROS, 4-HNE ↑	BAX ↑	Oxidative mtDNA (8-OHDG)	Ca2+ homeostasis ATP synthesis ↓ MMP depolarization	[9, 15, 16, 47]
Blue light	ROS, 4-HNE, MDA, HO-1, SOD1↑ GPX1 ↓	BAX ↑ Bcl-2 ↓ caspase-3 ↑	Oxidative mtDNA(8-OHDG)	ATP synthesis ↓	[117–119, 121, 209–211]
Sjögren’s syndrome	ROS, 4-HNE, HEL ↑	BAX ↑ Bcl-2 ↓ caspase-3 ↑	mtDNA release	mitochondria morphological changes, AMA mitophagy	[11, 127, 212]
Metabolic disease					
High-fat diet	ROS, NOX4, 3NT↑ malondialdehyde ↑ catalase ↑	BAX ↑	mtDNA release	Mfn2 ↓ Fis1, DRP1 ↑	[139–141, 213]
Diabetes	ROS, 4-HNE, 3NT↑	BAX ↑ Bcl-2 ↓	mtDNA damage mtDNA release	mitochondrial dynamics mitophagy Ca2+ homeostasis	[147, 149, 214–216]
Aging	ROS, HEL, 4-HNE ↑	caspase-3 ↑	8-OHdG		[153, 154]
Benzalkonium Chloride	ROS ↑	BAX ↑ Bcl-2 ↓Cyt-c ↑		ATP synthesis ↓	[73, 158, 159]

#### Blue light

DE has been increasingly associated with the prolonged use of electronic devices, particularly computers and smartphones. Research indicates that nearly half (49.5%) of individuals who frequently engage with digital screens worldwide experience symptoms of DE [112]. The primary light source in modern screens, including those of computers and mobile phones, is the light-emitting diode (LED) [113]. Notably, blue light, which is a significant optical component emitted by LEDs, is characterized as a high-energy visible light with wavelengths ranging between 400 and 500 nm [114]. This specific range of light is implicated in contributing to the discomfort and potential health risks associated with DE.

While the exact mechanisms underlying blue light-induced DE remain partially understood, a growing body of research highlights the significant impact of blue light exposure on cellular health. Blue light has been shown to trigger the production of ROS within cells, leading to oxidative damage [115–117]. This type of light exposure is particularly detrimental to mitochondrial health, manifesting as compromised mitochondrial ultrastructure, diminished rates of ATP synthesis, accumulation of ROS, and ultimately, cell death. Experimental evidence from studies where mice were exposed to equivalent doses of red, blue, and green light for ten consecutive days revealed that symptoms of ocular distress, such as increased corneal staining and reduced tear film breakup time, were uniquely observed following blue light exposure. These findings suggest a strong link between blue light exposure and oxidative damage, inflammation, and programmed cell death [118]. Further supporting this notion, studies involving human corneal and conjunctival epithelial cells have demonstrated that blue light irradiation directly contributes to the accumulation of ROS within these cells [119]. Excessive reactive oxygen species (ROS) in the cornea not only elevate the production of O_2_^−^ in mitochondria and impair mitochondrial function [120], but also trigger the release of inflammatory cytokines and recruitment of macrophages. Moreover, conjunctival cells appear to be more susceptible than corneal cells. While the cornea possesses a robust antioxidative defense mechanism—blue light induces the upregulation of SOD1 in corneal epithelial cells, providing protective effects—this upregulation of SOD1 is not observed suddenly. Furthermore, the expression of GPX1 is downregulated in conjunctival epithelial cells, heightening the risk from blue light exposure [121]. Considering blue light as a risk factor for DE, it is important to recognize the conflicting evidence regarding its pathogenic effects. While in vitro and animal studies have shown that blue light can induce oxidative stress and mitochondrial dysfunction, clinical trials [122, 123], such as the one by Singh et al. [124], have found that blue light blocking lenses do not alter the signs or symptoms of eye fatigue caused by computer use. This discrepancy highlights the need for further research to understand the specific conditions under which blue light may contribute to DE pathogenesis and to identify patient populations that might benefit from blue light filtering interventions.

### Sjögren’s syndrome

Sjögren’s syndrome, an autoimmune disorder primarily affecting exocrine glands, is marked by lymphocyte infiltration within these glands. This condition predominantly impacts the salivary and lacrimal glands, leading to symptoms such as dry mouth and DEs [125]. While Sjögren’s syndrome is classified as an autoimmune disease, emerging research points to a significant role of mitochondrial dysfunction in its pathogenesis. Historically, the presence of anti-mitochondrial antibodies has been noted across various autoimmune diseases, suggesting an underlying mitochondrial component [126, 127]. Specifically, in the context of Sjögren’s syndrome, patients’ salivary glands have shown signs of mitochondrial distress, including swelling of the mitochondrial matrix, loss and disintegration of cristae, and rupture of the mitochondrial membrane [11]. These mitochondrial anomalies contribute to an increase in ROS accumulation, leading to a cascade of pathological changes. A pivotal aspect of mitochondrial dysfunction in Sjögren’s syndrome is the elevation of mitochondrial ROS levels, accompanied by the release of mtDNA [11, 55]. This process triggers the activation of the NLRP3 inflammasome, culminating in the secretion of pro-inflammatory cytokines such as IL-18 [88]. Given these findings, some researchers advocate for recognizing mitochondrial dysfunction as a central inflammatory driver in Sjögren’s syndrome [128, 129]. The potential of targeting mitochondrial dysfunction as a therapeutic strategy has been underscored by studies on Metformin, an AMPK agonist. Metformin has demonstrated efficacy in enhancing salivary gland function in Sjögren’s syndrome patients by modulating mitochondrial activity [130]. This evidence positions mitochondria as a promising therapeutic target for addressing the underlying mechanisms of Sjögren’s syndrome and improving patient outcomes.

### Metabolic disease

As societies have progressed, with notable improvements in living standards and quality of life, there has been a marked shift in dietary habits towards energy-dense foods rich in fats and sugars. Coupled with a decline in physical activity—attributable to the sedentary nature of many modern occupations, changes in modes of transportation, and the rise of urban living—this shift has led to a significant energy imbalance. This imbalance, characterized by a surplus of calories consumed over calories expended, is a key driver behind the increasing rates of overweight and obesity observed globally. The World Health Organization (WHO) highlighted a concerning trend in its 2016 report, noting that the global prevalence of obesity had almost tripled since 1975. By 2016, nearly 2 billion adults worldwide, or 39% of the adult population, were classified as overweight [131, 132]. Among these individuals, approximately 650 million, or 13%, were considered obese. This dramatic rise in overweight and obesity rates underscores the urgent need for public health interventions aimed at addressing this growing epidemic. The consequences of excessive energy intake extend far beyond weight gain, imposing a significant burden on various bodily organs and increasing the risk of numerous diseases. Conditions such as type 2 diabetes, fatty liver disease, hypertension, myocardial infarction, stroke, dementia, osteoarthritis, obstructive sleep apnea, and various forms of cancer are all associated with overweight and obesity [133–136].

#### High-fat diet

Recent research has elucidated the detrimental effects of a long-term high-fat diet on ocular health, particularly in relation to. This dietary pattern has been linked to several adverse changes within the eye, highlighting the intricate relationship between diet, mitochondrial health, and ocular function. One of the primary findings is that a long-term high-fat diet contributes to lipid accumulation in the lacrimal gland, accompanied by mitochondrial hypertrophy. These changes disrupt the normal functioning of the lacrimal gland, leading to a reduction in tear production, a key factor in the development of DE symptoms [137]. Additionally, such a diet has been shown to negatively affect the Meibomian glands, which play a crucial role in maintaining tear film stability [138]. The loss of these glands, along with duct blockages, increased oxidative stress, and heightened inflammatory responses, culminates in Meibomian gland dysfunction—a common contributor to DE disease. Moreover, a high-fat diet exacerbates oxidative stress in ocular surface epithelial cells, increasing the expression of enzymes like NOX4 and markers of nitrosative stress such as 3-nitrotyrosine (3-NT) [139]. These molecular changes lead to damage resembling DE on the ocular surface. The underlying mechanisms involve an increase in circulating free fatty acids and their abnormal deposition in tissues, necessitating their breakdown through mitochondrial β-oxidation [140]. A diet high in fats overburdens this metabolic pathway, resulting in excessive production of ROS. The accumulation of ROS not only causes mitochondrial oxidative damage but also triggers the release of inflammatory factors and activates apoptotic pathways within mitochondria [141]. Crucially, a high-fat diet disrupts mitochondrial homeostasis, tipping the balance between the dynamic processes of mitochondrial fusion and fission. Proteins such as mitofusins 1 and 2 (Mfn1 and Mfn2), optic atrophy gene 1 (OPA1), dynamin-related protein 1 (Drp1), and fission protein 1 (Fis1) are key regulators of these processes [142]. A long-term high-fat diet has been associated with a decrease in Mfn2 expression and an increase in Fis1 and Drp1 expression, signaling mitochondrial dysfunction [143]. In summary, the consumption of a long-term high-fat diet leads to mitochondrial dysfunction and elevated levels of oxidative stress, adversely affecting various ocular surface tissues. These changes collectively contribute to the development of DE, underscoring the importance of dietary choices in maintaining ocular health and preventing DE.

#### Diabetes

Over the last two decades, diabetes has emerged as a significant global health issue, affecting approximately 463 million adults worldwide [144]. Among the myriad complications associated with diabetes, ocular issues, particularly diabetic retinopathy—a leading cause of blindness—are prominent. Additionally, diabetes has been recognized as a risk factor for various ocular surface lesions, including DE and diabetic keratopathy [145], further establishing it as a notable cause of DE [5]. Research indicates that over half (54.3%) of individuals diagnosed with type 2 diabetes experience symptoms of DE, underscoring the profound impact of this metabolic disorder on ocular health [146].

A key aspect of the connection between type 2 diabetes and ocular complications, including DE, lies in mitochondrial dysfunction. Type 2 diabetes is characterized by alterations in mitochondrial dynamics, OXPHOS, mitophagy, and calcium homeostasis, all of which contribute to the disease’s complexity [147–149]. Specifically, high glucose levels are known to induce mitochondrial dysfunction across various tissues and organs, a condition that is also linked to the development of diabetic DE. Early stages of this condition have been associated with a reduction in tear production and lacrimal gland weight, alongside decreased oxygen consumption rates (OCR) and basal extracellular acidification rates (ECAR). Furthermore, increased mtDNA damage has been observed, with the mitochondrial bioenergetic metabolism of the lacrimal glands being significantly more impaired than that of the corneal epithelium. These findings suggest that the susceptibility of lacrimal gland bioenergetics deficiency may precipitate the early onset of DE in diabetic individuals. Interestingly, the administration of mitochondria-targeted antioxidants, such as SkQ1, has shown promise in alleviating the severity of DE by improving mitochondrial function [150]. This suggests that targeting mitochondrial dysfunction could be a viable therapeutic strategy for managing diabetic DE. The potential of mitochondria-targeted antioxidants highlights an innovative approach to treating ocular complications associated with diabetes, offering hope for improved outcomes in affected individuals.

### Aging

Aging is a significant factor influencing the prevalence and severity of DE, with studies indicating a marked increase in its occurrence among individuals over the age of fifty [3]. This relationship between aging and DE can be attributed to various physiological changes that occur as part of the aging process, affecting the ocular surface and its associated glands. In aged mice, research has demonstrated several key changes that contribute to the development of DE, including disruption of corneal barrier function, heightened ocular surface inflammation [151], loss of meibomian glands [152], and increased levels of oxidative stress within the lacrimal glands [153, 154]. These findings in animal models mirror the clinical observations in humans, where similar age-related ocular changes contribute to DE symptoms. Interestingly, the relationship between aging and DE appears to be bidirectional, with DE conditions also promoting cellular senescence. Hyperosmotic stress, a condition commonly associated with DE due to tear film instability, has been shown to increase the expression of senescence markers such as p53, p21, and p16 [155]. This suggests that not only does aging predispose individuals to DE, but the presence of DE can further accelerate the aging process in ocular tissues.

A central feature of aging at the cellular level is the increased production of ROS, largely stemming from mitochondrial dysfunction. As mitochondria age, they become less efficient and more prone to releasing ROS, leading to oxidative damage [82]. This accumulation of ROS disrupts cellular signaling pathways and structural integrity [156], contributing to the aging process and the development of age-related diseases, including DE.

### Benzalkonium chloride (BAC)

BAC is the most widely used preservative in ophthalmic solutions, including eye drops. Its primary role is to prevent microbial contamination during the shelf life and use of these products. Despite its antimicrobial efficacy, the long-term application of eye drops containing BAC has been associated with an increased risk of developing DE [157]. This adverse effect can be attributed to BAC’s impact on ocular surface health and underlying cellular mechanisms. Research has demonstrated that BAC exerts a concentration-dependent inhibitory effect on mitochondrial functions, specifically targeting mitochondrial complex I. This inhibition leads to a significant reduction in mitochondrial ATP production (with an IC50 of 5.3 μM) and oxygen consumption (with an IC50 of 10.9 μM), effectively impairing over 90% of mitochondrial function at certain concentrations [158]. The disruption of mitochondrial activity by BAC has profound consequences for cellular health, primarily through the induction of oxidative stress [159]. The increase in cellular oxidative stress due to BAC exposure results from an imbalance between the production of ROS and the cell’s ability to detoxify these reactive intermediates or repair the resulting damage. This oxidative stress, in turn, triggers a cascade of detrimental cellular responses, including the release of pro-inflammatory factors [160]. Moreover, BAC exposure activates the mitochondrial apoptotic pathway, a form of programmed cell death that plays a crucial role in maintaining tissue homeostasis by eliminating damaged or dysfunctional cells. The activation of this pathway is characterized by the upregulation of pro-apoptotic proteins such as Bax, inhibition of anti-apoptotic proteins like Bcl-2, and the release of Cyt-c from mitochondria into the cytosol [73, 161]. These molecular events lead to the execution phase of apoptosis, contributing to cell loss and tissue damage on the ocular surface, thereby aggravating DE symptoms.

## Therapies targeting mitochondrial dysfunction

### Antioxidant therapy

#### Nrf2 activator

Nuclear factor erythroid 2-related factor 2 (Nrf2) plays a pivotal role as a cellular antioxidant regulator, orchestrating the expression of various antioxidant response element-dependent genes. This regulation is crucial for modulating both the physiological and pathophysiological outcomes following exposure to oxidants [162]. Consequently, Nrf2 emerges as a promising antioxidant therapeutic target in the realm of DE treatment.

Research has illuminated the therapeutic potential of Nrf2 modulation in addressing DE conditions. By promoting the expression of antioxidant enzymes, such as HO-1 and GPX1, Nrf2 activation enhances cellular defense mechanisms against oxidative stress. This upregulation leads to increased ATP levels and mitochondrial membrane potential, contributing to cellular energy balance and stability. Moreover, Nrf2 activation plays a crucial role in mitigating apoptosis, primarily by inhibiting the release of Cyt-c, a key component in the apoptotic pathway. Beyond its direct antioxidant effects, Nrf2 also significantly impacts cellular resilience against oxidative damage. Enhanced Nrf2 expression has been shown to reduce intracellular ROS and diminish 8-OHdG-stained cytogenesis, markers of oxidative DNA damage [163, 164]. Furthermore, Nrf2’s regulatory capacity extends to inhibiting the ROS-NLRP3-IL-1β signaling axis, a pathway implicated in inflammatory responses. By curtailing this axis, Nrf2 offers protection against hyperosmotic stress (HS)-induced cellular damage, highlighting its integral role in combating oxidative stress and inflammation associated with DE [165].

#### Vitamin supplementation

Vitamins, as natural substances with antioxidant properties, have garnered significant attention for their potential role in managing DE. Numerous studies have provided evidence supporting the benefits of vitamin supplementation in the context of ocular health and DE treatment [166, 167]. One area of interest revolves around vitamin D, which has been linked to cellular responses under hypertonic conditions. Research has indicated that the levels of vitamin D and its receptor (VDR) in human corneal epithelial (HCE) cells decrease in response to hypertonic stress, suggesting a potential involvement of vitamin D in this physiological process. Furthermore, studies have demonstrated that resveratrol (RES), a polyphenolic compound with antioxidant properties, can protect against hyperosmotic stress-induced ROS production by activating Notch signaling, ultimately leading to the restoration of vitamin D levels. This interplay between RES, Notch signaling, and vitamin D highlights the complex regulatory mechanisms implicated in ocular surface health and DE pathophysiology [168]. In clinical investigations, the use of eye drops containing 0.15% hyaluronic acid and vitamin B12 has shown promising results in reducing oxidative stress levels and improving various clinical parameters associated with DE, including the Ocular Surface Disease Index (OSDI) and fluorescein tear breakup time (BUT) [57]. The observed improvements in Schirmer’s test and tear film breakup time, along with the reduction in oxidative stress, underscore the therapeutic potential of vitamin B12 in managing DE symptoms.

#### SKQ1

SKQ1, a mitochondria-targeting antioxidant, has shown promising therapeutic potential in the treatment of various age-related eye diseases, including cataracts, AMD, glaucoma, and DE [169, 170]. Research has indicated that SKQ1 can enhance the antioxidant activity and stability of the tear film, protecting against ROS-induced apoptosis and inflammatory responses. By mitigating these harmful processes, SKQ1 can help maintain ocular surface health and alleviate DE symptoms [171]. In an age-related DE model, SKQ1 has been found to attenuate aging-induced ultrastructural changes and restore mitochondrial numbers in the lacrimal gland. This suggests that SKQ1 may have a positive impact on cellular and tissue health in the context of DE associated with aging [172]. In the DE rats induced by benzalkonium chloride, the newly developed mitochondrial-targeted SkQ1 nanoparticles exhibited significantly higher activity in scavenging cytosolic and mitochondrial ROS. Additionally, they effectively suppressed the activation of the NLRP3 inflammasome and the inflammatory signaling mediated by NLRP3 [173]. Furthermore, SKQ1 has demonstrated improvements in diabetic DE by enhancing mitochondrial function [150]. Diabetes can adversely affect ocular health, leading to DE symptoms. SKQ1’s ability to improve mitochondrial function may contribute to its efficacy in managing DE in diabetic individuals. Clinical studies evaluating the safety and effectiveness of SKQ1 in DE treatment have reported positive outcomes. Topical application of SKQ1 eye drops 2–3 times per day for 4–6 weeks has been shown to increase tear film stability, reduce corneal fluorescein staining, and alleviate DE symptoms such as dryness, burning, foreign body sensation, and blurred vision [17, 174].

#### Anti-senescence drugs

Various age-related diseases are associated with decreased secretion of melatonin, suggesting an important role for melatonin in age-related diseases [175]. Melatonin has been shown to play important roles in age-related eye diseases such as glaucoma [176] and AMD [177]. Many studies have demonstrated the role of melatonin in DE. Melatonin can reduce excessive ROS production and LDH release and maintain mitochondrial function by activating HO-1 expression, reducing apoptosis and inflammatory cascades [178]. Melatonin and its derivatives can promote tear secretion by interacting with different melatonin receptors [179, 180].

Astaxanthin is a lutein carotenoid and is the most abundant carotenoid in marine life. Astaxanthin neutralizes free radicals and other oxidants by accepting or donating electrons without being destroyed or becoming a pro-oxidant in the process, with anti-senescence potential [181, 182]. Pretreatment with astaxanthin liposomes can reduce ROS generation in corneal epithelial cells and significantly inhibit the expression of the aging-related genes P53, P21, and P16 in in vivo and in vitro DE models [155, 183].

#### Other antioxidants

According to the key role of oxidative stress in the pathogenesis of DE, many antioxidants targeting ROS production have shown the potential of DE treatment. Eurya japonica [66], Camellia japonica [184], taurine [185], Chamaecyparis obtuse [186], KR-67607 [160], and Zidovudine [187] downregulated ROS generation in DE to reduce oxidative damage and inflammatory responses (Table 2).

**Table 2:** Targeting to the mitochondria for treating DE.

Treatment	DE model	Mechanism	Effect	Ref.
Edaravone	Hyperosmotic Stress	Nrf2	HO-1 ↑ GPX1 ↑ ATP ↑ ROS ↓ Cytochrome C ↓ NLRP3/IL-1β ↓	[163]
Esculetin	H_2_O_2_-treated	Nrf2		[164]
Calcitriol	Hyperosmotic Stress	Nrf2		[165]
RES	Hyperosmotic Stress	Notch/VDR	Vitamin D ↑ ROS ↓	[168]
Vitamin B12	Dry Eye Patients		ROS ↓ TBUT ↑ Tear production ↑ OSDI ↓	[57]
SKQ1	Ageing Hyperglycemia Dry Eye Patients		Mitochondria ↑ TBUT ↑ Corneal fluorescein staining scores ↓ Symptom ↓	[17, 150, 172, 174]
Melatonin/ Melatonin derivative	H_2_O_2_-treated	HO-1	ROS ↓ LDH ↓ Aopotosis ↓ Inflammation ↓ Tear production ↑	[178–180]
Astaxanthin	Desiccating Stress Lacrimal Glands Extraction		ROS ↓ P53,P21,P16 ↓	[155, 183]
Eurya japonica	H_2_O_2_-treated Desiccating Stress		ROS ↓ Mitochondrial Depolarization ↑ TNF-α, IL-1β, IP-10 ↓ Tear production ↑ Corneal fluorescein staining scores ↓	[66]
Camellia japonica	H_2_O_2_-treated desiccating stress		ROS ↓ Mitochondrial Depolarization ↑ MnSOD, Catalase ↑ Corneal fluorescein staining scores ↓ IFN-γ, IL-1β, IL-6, TNF-α ↓	[184]
N-acetylcysteine (NAC)	Desiccating stress 4-HNE-induced		ROS ↓ Corneal fluorescein staining scores ↓ NLRP3/IL-1β ↓	[63, 217]
Taurine	H_2_O_2_-treated Atropine-induced		ROS ↓ Tear production ↑ MMP-9 ↓	[185]
Chamaecyparis obtusa	H_2_O_2_-treated desiccating stress		ROS ↓ HO-1, Catalase ↑ Tear production ↑ Corneal fluorescein staining scores ↓	[186]
KR-67607	Benzalkonium chloride (BAC)-induced		4-HNE ↓ SOD1 ↑ IL-6, TNF-α ↓	[160]
Zidovudine	Hyperosmotic stress		ROS ↓ SOD1 ↑ IL-6, NF-kB ↓	[187]
L-Carnitine	Hyperosmotic stress		ROS, MDA, 4-HNE, 8-OHdG ↓ SOD1, GPX1, PRDX4 ↑	[46]
SP	Hyperosmotic stress	Akt/NK-1	Ca2^+^ ↑ ROS↓ glutathione ↑ Apoptosis ↓	[218]
α-MSH/ CMC	Hyperosmotic stress Desiccating stress		ROS↓ NLRP3 ↓ FL ↓ Goblet cells ↑ Tear production ↑	[219]
cHA-Cr-L	Hyperosmotic stress		ROS ↓ IL-1β, TNFα ↓	[220]
Pterostilbene	Hyperosmotic stress		ROS, MDA, 8-OHdG, COX2 ↓ SOD1, PRDX4 ↑ TNF-α, IL-1β, IL-6, MMP-2, MMP-9 ↓	[221]
BSP	BAC-induced lipopolysaccharide-induced		ROS ↓ TNFα, IL-8 ↓ Tear production ↑	[222]
Medicinal Plant Extracts	Desiccating stress Dry eye patients		4-HNE, ROS ↓ IL-1β, IL-6, TNF-α, IFN-γ ↓ CXCR3^+^ T ↓ Goblet cells ↑ OSDI ↓ BUT ↑	[223, 224]
Xanthan gum	H_2_O_2_-treated		ROS ↓	[225]
Epigallocatechin gallate	Hyperosmotic Stress IL-1β-induced		ROS ↓pMAPK, pJNK, NFκB ↓	[226]

### Anti-mitochondrial apoptosis

Cyclosporine A (CsA) is widely recognized as a prevalent immunosuppressive agent in the management of DE. Its therapeutic action extends beyond the mere suppression of immune cell activation, delving into the realm of mitochondrial function enhancement. A pivotal aspect of CsA’s mechanism involves its direct interaction with cyclophilin D (CypD) located on the mitochondrial membrane. CypD plays a crucial role as a component of the mitochondrial permeability transition pore (mPTP), a complex that is essential for regulating mitochondrial membrane permeability and, consequently, cellular health.

The inhibition of mPTP by CsA has significant repercussions for cellular apoptosis pathways. Specifically, this inhibition leads to a reduction in the expression of Bax, a pro-apoptotic protein, and an increase in the expression of Bcl-2, an anti-apoptotic protein. The balance between these two types of proteins is critical for determining the cell’s fate, particularly in the context of apoptosis. By tipping the scales in favor of anti-apoptotic signals, CsA helps to mitigate mitochondrial apoptosis, a process that contributes to cell death and tissue damage in DE conditions.

Empirical evidence supporting the efficacy of CsA in this domain comes from studies investigating its application in DE treatment. One notable study demonstrated that topical application of CsA for a duration of 7 days led to a marked amelioration of mitochondrial apoptosis. This finding not only underscores the potential of CsA to improve cellular survival and integrity by modulating mitochondrial dynamics but also highlights its therapeutic value in addressing the underlying cellular disturbances associated with DE.

### Calorie restriction (CR)

CR is clearly defined as a reduction in energy intake well below the amount of calories that would be consumed ad libitum (≥10% in human studies, usually ≥20% in rodents) [188]. This is a nutritional intervention that reduces energy intake while maintaining nutrient adequacy and has been shown to prolong healthy lifespan in rodent and primate models [189]. Substantial evidence has shown that CR benefits chronic health problems such as type 2 diabetes [190], senescence [191], cancer [192], cardiovascular disease [193, 194] and inflammatory diseases [195, 196]. CR can activate numerous endocrine and neurobiological responses ranging from the systemic level to molecular signaling pathways. Several hypotheses have been proposed regarding the mechanism by which CR promotes health, including the stress resistance hypothesis, the oxidative stress hypothesis, the altered glucose and insulin hypothesis, and the growth hormone/insulin-like growth factor-1 axis hypothesis [197, 198].

Several studies have reported the therapeutic effect of CR on age-related eye diseases [199], such as cataracts [200], uveitis [201], glaucoma [202], and fundus diseases [203–207]. Since aging and oxidative stress increase the risk of developing DE, CR is also considered to have a possible therapeutic potential for DE. After 6 months of CR, the tear volume significantly improved, and histochemical staining showed a higher density of lacrimal acinar units, a lower degree of interstitial fibrosis and reduced oxidative stress (8-OhdG and 4-HNE). Electron microscopy observation showed that the mitochondrial structure of the rat lacrimal gland was more complete after 6 months of CR. These results demonstrate that CR protects the mitochondrial structure in the lacrimal gland and mitigates oxidative stress-related damage while preserving lacrimal gland function [208].

## Conclusions

This study provides a comprehensive examination of the role of mitochondrial dysfunction in DE, delving into its underlying mechanisms and the therapeutic strategies currently targeting this dysfunction. It underscores the importance of further research into mitochondrial dynamics and functions to develop more effective treatments for DE, suggesting a potential shift in therapeutic paradigms towards addressing the mitochondrial dysfunctions that contribute to this widespread condition. Mitochondrial dysfunction has emerged as a crucial factor in the pathogenesis of various diseases, including DE, highlighting the complex relationship between DE and mitochondrial dysfunction. Numerous factors leading to DE, such as environmental stress, blue light exposure, autoimmune diseases, metabolic disorders, and aging, are closely linked to mitochondrial disturbances. Despite these links, the precise mechanisms by which mitochondrial dysfunction influences DE remain complex and not fully elucidated. This gap in understanding accentuates the need for additional research into how mitochondrial dysfunction affects the development and progression of DE. Current treatments primarily focus on alleviating oxidative stress caused by mitochondrial dysfunction, yet mitochondrial impairment can result in various types of damage. This review outlines how various etiological factors might lead to mitochondrial dysfunction, thereby contributing to the onset and exacerbation of DE. These dysfunctions include mitochondrial oxidative stress, apoptosis, and DNA damage. Interestingly, treatments targeting mitochondrial dysfunction have shown promising results, highlighting the importance of mitochondrial health as a critical area for therapeutic development.

## Data Availability

The datasets during and/or analyzed during the current study are available from the corresponding author on reasonable request.
